# Procedural Treatments as Adjuncts in the Management of Acne Vulgaris: A Narrative Review

**DOI:** 10.1111/jocd.70832

**Published:** 2026-04-10

**Authors:** Katarzyna Beutler, Danuta Nowicka, Michael H. Gold, Karolina Chilicka‐Hebel

**Affiliations:** ^1^ University Centre of General Dermatology and Oncodermatology Wroclaw Medical University Wroclaw Poland; ^2^ Division of Aesthetic Dermatology and Regenerative Medicine of the Skin Wroclaw Medical University Wroclaw Poland; ^3^ Gold Skin Care Center Tennessee Clinical Research Center Nashville Tennessee USA; ^4^ Department of Health Sciences, Institute of Health Sciences University of Opole Opole Poland

**Keywords:** acne vulgaris, adjunct treatment, cosmetic procedures

## Abstract

**Background:**

Acne vulgaris is a chronic inflammatory disorder of the pilosebaceous unit and one of the most prevalent dermatological conditions globally. Its multifactorial pathogenesis and variable clinical presentation necessitate a multimodal therapeutic approach. While pharmacological interventions remain first‐line therapy, cosmetic and dermatologic procedures are increasingly utilized as adjunctive strategies to enhance clinical outcomes. This narrative review synthesizes current evidence on four commonly employed cosmetic modalities in acne management: chemical peels, mechanical peels, light‐based therapies, and radiofrequency‐assisted microneedling.

**Aim:**

The aim of this study is to evaluate the role of procedural treatments as adjunctive options in the management of acne vulgaris.

**Methods:**

A literature search was performed in PubMed, Web of Science, and Scopus with no time restrictions, focusing on patients with acne vulgaris and adjunct cosmetic/procedural interventions.

**Results:**

Given the narrative scope, this was a non‐comprehensive, non‐exhaustive evidence synthesis, with treatment options organized thematically. Overall, these cosmetic procedures can provide meaningful adjunctive benefits when integrated into individualized acne treatment plans. However, variability in study designs, procedural parameters, and outcome measures limits direct comparison across modalities. Further high‐quality randomized controlled trials are necessary to establish standardized protocols and to determine long‐term safety and efficacy.

**Conclusions:**

Procedural treatments can serve as valuable adjuncts in the management of acne vulgaris by enhancing therapeutic outcomes and addressing treatment‐resistant cases. However, individualized treatment selection and further high‐quality studies are needed to optimize their use and long‐term benefits.

## 1 Introduction

Acne vulgaris is a chronic inflammatory disease that affects the hair follicles and sebaceous glands. Skin lesions most often affect the face and trunk. Its distinctive features include comedones, papules, pustules, and nodules. In more extreme situations, it may cause scarring, erythema, and discoloration. Although adult patients may also suffer from the development or continuation of the condition, adolescence is when it usually appears. Up to 80%–100% of people in the 11–30 age range are affected by this illness. Acne vulgaris, one of the most common skin disorders in the world, has a substantial impact on a person's social functioning, mental health, and quality of life. Depending on the severity of the condition, topical or systemic medication, or a combination of both, is used to treat it. Although they serve a supportive role, cosmetic operations have a substantial influence on the development and treatment of acne vulgaris [1, 2, 3].

## 2 Etiopathogenesis

Acne vulgaris is caused by four primary interconnected pathomechanisms: excessive sebum production caused by androgens, aberrant keratinization that blocks the hair follicle outlet, *Cutibacterium acnes* bacteria colonizing the skin, and inflammatory responses. Excessive keratinization of the hair follicle openings is caused by immune system cells that build up in the hair follicle and trigger the release of pro‐inflammatory cytokines. A comedone, a non‐inflammatory lesion, is created when overly keratinized epidermal cells and sebum obstruct the hair follicle entrance. In turn, the comedone may develop into inflammatory lesions like papules, pustules, or nodules due to the interaction of the hair‐sebaceous unit with the bacteria present there (mostly *Cutibacterium acnes*) and sebum [1, 4, 5, 6].

## 3 Cosmetic Treatments Used as an Adjunct in the Treatment of Acne Vulgaris

The function of cosmetic procedures in the acne treatment algorithm is clearly stated in the most recent guidelines. Physical therapies, including peels and light therapy are recommended as additional options, especially for scar removal and mild to moderate acne. In acne treatment algorithms, cosmetic treatments can be utilized in addition to medication; the patient's preferences, skin phototype, and acne phenotype should all be taken into consideration. Research is still needed to determine these methods' long‐term efficacy and safety [2, 7]. Cosmetic treatments affect the pathophysiology of acne vulgaris through physical, chemical, and photobiological processes, including excessive keratinization, sebum generation, *Cutibacterium acnes* colonization, and the inflammatory process [1].

Poor adherence to conventional acne therapies remains a major barrier to achieving sustained clinical control, particularly given the chronic and relapsing nature of the disease and the need for long‐term maintenance regimens. Real‐world evidence suggests that persistence with guideline‐recommended topical treatments is often limited; in a survival analysis of patients with moderate acne, the overall median duration of topical therapy use was only 2 months, with a 50% probability of discontinuation by 3 months, most commonly due to perceived ineffectiveness and treatment‐related tolerability issues [8]. Similar challenges have been reported in broader patient populations, with a large cross‐sectional survey in Thailand showing adherence in only one‐third of respondents, and indicating that many patients prefer combined approaches that frequently include procedural modalities, while oral treatments are comparatively underutilized [9]. In parallel, barriers to dermatologic care, including cost and access limitations, may further impact treatment decision‐making and contribute to reliance on alternative or non‐prescription acne treatments [10]. Together, these findings highlight the importance of adjunctive interventions that can align with patient preferences and improve real‐world treatment engagement. Therefore, the aim of this narrative review was to synthesize current evidence on four commonly employed cosmetic modalities in acne management: chemical peels, mechanical peels, light‐based therapies, and radiofrequency‐assisted microneedling.

This article was designed as a narrative review. A literature search was performed in PubMed, Web of Science, and Scopus, with no time restrictions applied. The population of interest comprised patients with acne vulgaris, and the review focused on cosmetic and procedural interventions used as adjunctive treatments. Given the narrative scope, the search was intended to identify representative and clinically relevant publications rather than to provide a comprehensive or exhaustive evidence synthesis. The included treatment options were organized thematically, and the available findings were summarized and interpreted in the context of clinical practice to reflect the authors' expert perspective [11].

### 3.1 Chemical Peels

Acne vulgaris is treated using chemical peels based on salicylic acid (BHA), glycolic acid (AHA), azelaic acid, pyruvic acid, lactic acid, mandelic acid, Jessner's solution, and trichloroacetic acid (TCA). Due to its favorable safety profile and ability to effectively reduce acne lesions, superficial peels are most frequently utilized. While Jessner's solution and TCA (especially at a dosage of 25%) are linked to a greater risk of problems, including post‐inflammatory hyperpigmentation (PIH), salicylic acid (30%) and glycolic acid offer the best effectiveness and favorable safety profile. Chemical peels unclog hair follicles, lessen blackheads, and restore normal keratinization by carefully exfoliating the stratum corneum. While glycolic and pyruvic acids acidify the environment and break down bacterial cell membranes, killing *Cutibacterium acnes*, salicylic acid also possesses anti‐inflammatory and sebostatic qualities. Sebum production is decreased by azelaic and pyruvic acids, which also lessen erythema and post‐inflammatory hyperpigmentation. The patient's tolerance, acne phenotype, and skin phototype all influence which peel is best. 8 According to extensive historical research, the majority of peel side effects are temporary (scabs, erythema, post‐inflammatory hyperpigmentation) and go away in a few weeks to months. Active skin infections, open wounds, pregnancy (for some compounds), recent isotretinoin usage (up to 6–12 months after the conclusion of therapy) a propensity for scarring (keloids) and hypersensitivity to peel ingredients are among the contraindications and risk factors. PIH and scarring are considerably more common in patients with higher skin phototypes (Fitzpatrick IV–VI), particularly following medium‐depth and deep peels. Superficial peels, lower concentrations, cautious qualification, and comprehensive pre‐ and post‐treatment care (including photoprotection and emollients) are advised for this population. Based on studies, those with phototype VI are more than five times more likely to have negative impacts than those with lower phototypes [12, 13, 14, 15].

### 3.2 Mechanical Peels

One kind of mechanical exfoliation is microdermabrasion. When treating mild to severe cases of acne vulgaris as well as acne scars, microdermabrasion is a supportive treatment. Using a stream of tiny crystals (such as aluminum oxide) and negative pressure, the mechanism of action involves controlled, superficial exfoliation of the stratum corneum, which improves skin texture, reduces blackheads, and unblocks hair follicle outlets. Furthermore, microdermabrasion can improve the benefits of pharmacological therapy by increasing the penetration of topical drugs into the epidermis. Microdermabrasion is typically used as an adjunct to topical or systemic treatment since its efficacy as a monotherapy is moderate. While the impact on inflammatory lesions is minimal, clinical trials have demonstrated improvement in non‐inflammatory lesions (comedones) and minor acne scars. Active bacterial, viral, or fungal skin infections, open wounds, severe inflammatory acne, a propensity for scarring (keloids), healing difficulties, recent surgery in the treatment region, and anticoagulant usage are among the conditions that exclude microdermabrasion. Data from multicenter trials demonstrate that, in contrast to mechanical dermabrasion, microdermabrasion is safe for patients undergoing or just finished isotretinoin medication, especially in higher skin phototypes [16, 17, 18].

### 3.3 Light Therapies

Non‐ablative lasers (like Nd:YAG), intense pulsed light (IPL), blue/red light, photodynamic treatments (PDT) with aminolevulinate or methylaminolevulinate, selective photothermolysis, and fractional CO2 lasers are some of the types of light therapy used to treat acne vulgaris [19]. Both inflammatory and non‐inflammatory lesions, as well as acne scars, are treated with these treatments.

Porphyrins produced by *Cutibacterium acnes* are activated by blue light (about 415 nm) and red light (about 630 nm), which results in the production of reactive oxygen species, the selective killing of bacteria, and a decrease in inflammation. Applying a photosensitizer (such as ALA or MAL) that, when triggered by blue or red light, causes toxicity to the sebaceous glands and bacteria results in a decrease in sebum production and the quantity of inflammatory lesions. This process is known as PDT. The skin microbiota is impacted by PDT. In addition to decreasing the quantity of *Staphylococcus epidermidis* and *Cutibacterium acnes*, it also lowers the skin's total microbial diversity. The method of action of PDT, which includes the selective elimination of bacteria within the hair‐sebaceous unit, is linked to this effect [3, 7, 20, 21]. IPL and lasers (Nd:YAG, CO_2_) cause thermal damage to sebaceous glands, reduce blood vessels around inflammatory lesions, and promote skin remodeling. Active skin infections, a propensity for scarring (keloids), pregnancy (for PDT), the use of photosensitizing medications, autoimmune skin conditions, recent surgery in the treatment area, and higher skin phototypes (IV–VI) because of the increased risk of post‐inflammatory hyperpigmentation are all contraindications for light therapy. Erythema, discomfort, burning, post‐inflammatory hyperpigmentation, and, less often, scarring and infections are the most frequent adverse effects. The kind of device, the settings of the treatment, and the unique qualities of each patient all affect efficacy and safety [3, 7, 21]. Novel laser technologies targeting sebaceous glands are emerging as promising adjuncts in acne management, particularly the 1726‐nm laser, which is designed to induce selective photothermolysis while preserving surrounding skin structures. Preclinical modeling and histologic data support its gland‐selective thermal effect when combined with contact cooling, potentially improving safety and tolerability in clinical use [22]. Early clinical evidence also suggests meaningful reductions in inflammatory lesions with mostly mild, transient adverse events and good patient satisfaction, supporting continued interest in this modality for moderate‐to‐severe acne [23, 24].

### 3.4 Microneedle Radiofrequency

In order to treat acne vulgaris, insulated microneedles are inserted into the dermis. The radiofrequency energy they emit selectively damages the sebaceous glands, decreases the production of sebum, and causes skin remodeling by promoting neocollagenesis and raising the expression of TGF‐β and type I collagen. Additionally, it has an anti‐inflammatory impact by lowering the production of inflammatory mediators including IL‐8 and NF‐κB, which results in fewer inflammatory and non‐inflammatory lesions. Clinical testing has proven the decrease of sebum and acne lesions, and the therapeutic impact lasts for several months. Active skin infections (bacterial, viral, or fungal), open wounds, a propensity for scarring (keloids), severe healing disorders, uncontrolled diabetes, use of anticoagulants, recently finished isotretinoin therapy (a minimum 6‐month break is advised), pregnancy, autoimmune skin diseases, and implanted electronic devices (such as pacemakers) are among the conditions that preclude the use of radiofrequency microneedling. Although the therapy is thought to be safe for those with higher skin phototypes, caution should be used because of the possibility of post‐inflammatory hyperpigmentation. Temporary erythema, swelling, discomfort, pinpoint bleeding, and, seldom, post‐inflammatory hyperpigmentation are the most frequent adverse effects. Typically, side effects are brief and modest [25, 26, 27].

## 4 Conclusions

Cosmetic procedures vary in their efficacy. Salicylic acid peels (reported reductions up to ~85%) and glycolic acid peels (up to ~80%) appear among the most effective options for reducing both inflammatory and non‐inflammatory acne lesions. Pain and burning restrict the tolerance of PDT, which improves inflammatory lesions by a median of 40%–56%. Both inflammatory and non‐inflammatory lesions can be effectively treated with Nd:YAG lasers, CO_2_ fractional lasers and microneedle radiofrequency. It has a good safety profile and a reduced recurrence rate [28, 29]. Although microdermabrasion is safe and can cure minor acne and acne scars, its efficacy as a stand‐alone treatment is only moderate. When individual qualifications and contraindications are considered, cosmetic therapies are a useful addition to pharmaceutical treatment of acne vulgaris, particularly in mild and moderate forms and in scar therapy (Table 1) [1, 3, 30].

**TABLE 1:** Cosmetic procedures and efficiency [3, 12, 21, 26, 28, 29].

Cosmetic procedure	Inflammatory acne lesions	Non‐inflammatory acne lesions	Efficiency
Salicylic acid peel (30%)	no	yes	Preferred in comedonal acne; well tolerated; most effective in non‐inflammatory lesions (up to 85%)
Glycolic peel	no	yes	For comedonal acne, it is up to 80% effective and well‐tolerated.
Jessner peel, TCA 25%	no	yes	Reduced effectiveness; increased risk of PIH, particularly in higher skin phototypes; infrequent usage
Microdermabrasion	no	yes	Moderately effective; safe when used alone; supportive of non‐inflammatory alterations and scarring
Photodynamic therapy (PDT)	yes	no	Red light is most effective; limited tolerance (pain, burning sensation); median decrease of inflammatory alterations by up to 56%.
IPL (Intense Pulsed Light)	yes	no	Reduction in inflammatory alterations by 42%–62%; effectiveness verified in RCT; short‐term effects.
Nd:YAG laser	yes	yes	Reduction of up to 83%; fewer recurrences in non‐inflammatory lesions; effective in both inflammatory and non‐inflammatory lesions.
Fractional laser CO_2_	yes	yes	RCT‐confirmed reduction of inflammatory lesions by up to 88%; also effective on acne scars.
Microneedle radiofrequency	yes	yes	Reduced recurrence rate compared to laser therapy; favorable safety profile; effective in both inflammatory and non‐inflammatory alterations

## Author Contributions

K.B. wrote the paper; D.N., M.H.G., and K.C.‐H. were writing, review and editing the paper.

## Funding

The authors have nothing to report.

## Disclosure

All authors read and approved the final version of the manuscript.

## Conflicts of Interest

The authors declare no conflicts of interest.

## Data Availability

Data sharing not applicable to this article as no datasets were generated or analysed during the current study.
